# A truncated protein product of the germline variant of the *DUOX2* gene leads to adenomatous polyposis

**DOI:** 10.20892/j.issn.2095-3941.2020.0305

**Published:** 2021-02-15

**Authors:** Mengyuan Yang, Yingxin Zhao, Yuwei Ding, Juan Wang, Yinuo Tan, Dong Xu, Ying Yuan

**Affiliations:** 1 Department of Medical Oncology, Zhejiang University School of Medicine, Hangzhou 310009, China; 2 Cancer Institute, Zhejiang University School of Medicine, Hangzhou 310009, China; 3 Department of Colorectal Surgery and Oncology, Key Laboratory of Cancer Prevention and Intervention, Ministry of Education, The Second Affiliated Hospital, Zhejiang University School of Medicine, Hangzhou 310009, China

**Keywords:** Adenomatous polyposis, *DUOX2*, whole-exome sequencing, endoplasmic reticulum retention, unfolded protein response

## Abstract

**Objective:**

In some patients with adenomatous polyposis, an identifiable pathogenic variant of known associated genes cannot be found. Researchers have studied this for decades; however, few new genes have been identified.

**Methods:**

Adenomatous polyposis coli (*APC*) negative polyposis patients were identified through next-generation sequencing and multiplex ligation-dependent probe amplification. Then, whole-exome sequencing (WES) was used to determine candidate genes harboring pathogenic variants. Functional experiments were performed to explore their effects. Subsequently, using Sanger sequencing, we found other polyposis patients carrying variants of the *DUOX2* gene, encoding dual oxidase 2, and analyzed them.

**Results:**

From 88 patients with suspected familial adenomatous polyposis, 25 unrelated *APC* negative polyposis patients were identified. Based on the WES results of 3 patients and 2 healthy relatives from a family, the germline nonsense variant (c.1588A>T; p.K530X) of the *DUOX2* gene was speculated to play a decisive role in the pedigree in relation to adenomatous polyposis. During functional experiments, we observed that the truncated protein, hDuox2 K530, was overexpressed in the adenoma in a carrier of the *DUOX2* nonsense variant, causing abnormal cell proliferation through endoplasmic reticulum (ER) retention. In addition, we found two unrelated *APC* negative patients carrying *DUOX2* missense variants (c.3329G>A, p.R1110Q; c.4027C>T, p.L1343F). Given the results of the *in silico* analysis, these two missense variants might exert a negative influence on the function of hDuox2.

**Conclusions:**

To our knowledge, this is the first study that reports the possible association of *DUOX2* germline variants with adenomatous polyposis. With an autosomal dominant inheritance, it causes ER retention, inducing an unfolded protein response.

## Introduction

Familial adenomatous polyposis (FAP, OMIM 175100) is an autosomal dominant disorder in which hundreds to thousands of adenomatous polyps develop^1^. Depending on the severity of polyposis, patients are classified as either classic FAP (CFAP) or attenuated FAP (AFAP). Compared to CFAP, AFAP is characterized by a smaller number of polyps (10–99 polyps), a later age of onset, and a lower probability of malignant transformation of the adenomas^2^.

The germline variants in the tumor suppressor gene, *APC* (adenomatous polyposis coli), are widely recognized as the leading genetic cause of this disease. The mutation detection rate of the *APC* gene in patients with FAP is closely related to the phenotype, as it is up to 82% in CFAP patients, and only 24% in patients with AFAP^3^. This highlights that the genetic background of some of the FAP patients remains unclear, especially in people with attenuated phenotypes. We named these patients with polyposis without an identifiable *APC* pathogenic variant as *APC* negative polyposis. In the recent decades, several genes have been reported to play important roles in *APC* negative polyposis. Germline biallelic pathogenic variants in *MUTYH* (mutY DNA glycosylase) were detected in 17% of the individuals with *APC* negative polyposis, but no biallelic variant was noted in typical cases^4^.

For suspected patients of adenomatous polyposis, early genetic screening is necessary to identify disease-causing gene variants in order to guide the treatment, follow-up, and family management strategies^5^. For patients with *APC* negative polyposis, as the cause of the disease is unknown, individualized therapeutic strategy cannot be formulated. Over the decades, numerous studies have been conducted worldwide in an attempt to identify genes responsible for adenomatous polyposis. A vast majority of those studies still focused on the *APC* gene, and very few new genes have been found. Therefore, we conducted a multi-center clinical study of the Chinese population to collect pedigrees of *APC* negative polyposis, aiming to find a novel disease-causing gene for adenomatous polyposis.

## Materials and methods

### Patients and DNA samples

**Figure 1:**
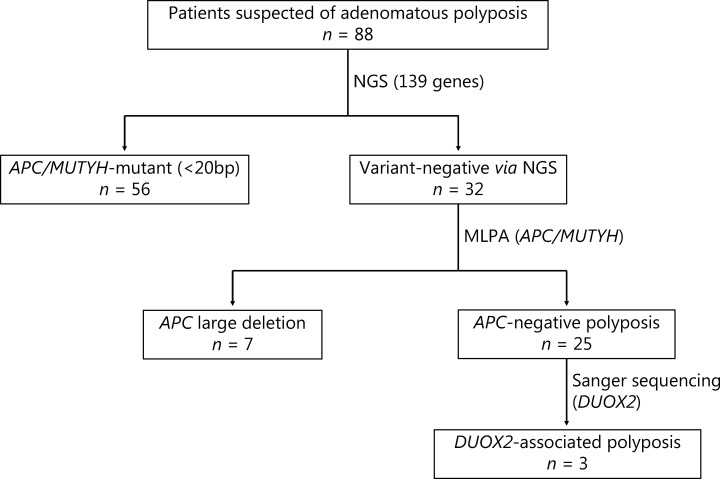
Flow chart of the multi-center clinical study. Patients enrolled in this study underwent multiple genetic tests to identify germline pathogenic variants. NGS, next-generation sequencing; MLPA, multiplex ligation-dependent probe amplification.

In this study, patients suspected of adenomatous polyposis were included. The criteria used were more than 10 polyps observed under colonoscopy, and pathological confirmation of adenoma. Clinical data and pedigree information were collected. The protocol of this study is shown in **Figure 1**. The study was approved by the Ethics Committee of the Second Affiliated Hospital of Zhejiang University School of Medicine (Approval No. 2017.066). Written informed consent was obtained from all patients.

Genomic DNA was extracted from peripheral EDTA-anticoagulated blood samples using the standard salting-out procedure (QIAamp DNA blood midi kit; Qiagen, Hilden, Germany). For pedigree members, if blood samples were not available, DNA was extracted from oral swabs using a TIANamp swab DNA kit (Tiangen, Beijing, China) or the negative margins of surgical specimens using a QIAamp DNA FFPE tissue kit (Qiagen). High quality DNA (OD260/280 = 1.8–2.0) samples were used for further genetic testing.

### Next-generation sequencing (NGS) and analysis

Variants of 139 genes associated with different hereditary cancers and polyposis were screened by NGS, which was performed by Genetron Health (Beijing, China) on the HiSeqX-ten sequencing platform (Illumina, San Diego, USA). These 139 genes are shown in **Supplementary Table S1**. We classified germline variants according to the American College of Medical Genetics and Genomics standards and guidelines for sequence variant interpretation^6^. *APC* mutant polyposis (FAP) was diagnosed if the patient was accompanied by germline pathogenic or likely pathogenic variants in the *APC* gene.

### Multiplex ligation-dependent probe amplification

Genomic DNA samples from patients without an identified pathogenic variant were examined for large deletions or duplications of *APC* and *MUTYH* genes using multiplex ligation-dependent probe amplification (MLPA) (SALSA^®^ MLPA^®^ probemix P043-E1; MRC, Amsterdam, The Netherlands) according to the manufacturer’s instructions. The results were analyzed against controls using Coffalyser software (MRC). A dosage quotient of 0.8–1.2 was interpreted as normal; 0, 0.4–0.65 and 1.3–1.65 were interpreted as homozygous deletion, heterozygous deletion or heterozygous duplication, respectively. For each sample, we performed 3 independent experiments.

### Whole-exome sequencing (WES)

WES was performed by XY Biotechnology (Shanghai, China) for genome analysis by capture using the Agilent SureSelect Human All Exon V6 (Agilent, Santa Clara, CA, USA). After capturing and enriching all exon regions, sequencing was performed on a HiSeqX-ten sequencing platform (Illumina). An average of 95% of all bases were covered 20 or more times. The quality control of raw exome data was conducted using fastp (version 0.14.1). Only high quality raw data (> 80% of the sequenced reads having a Qphred of 30) were subjected to further analysis. Sequenced reads were aligned to the GRCh37/hg19 human reference genome.

### Sanger sequencing

The variant NM_014080.4: c.1588A>T (p.K530X) of *DUOX2* was validated by Sanger sequencing according to standard protocols. Genomic DNA was used to amplify the corresponding region (primers: 5′-CCCCTCTGGGTCTCTTTTCT-3′ and 5′-TTGATCCTTCTCTGGCCACT-3′). PCR products were purified and sequenced on an ABI 3730 DNA Analyzer (Applied Biosystems, Foster City, CA, USA). We designed 21 pairs of primers (**Supplementary Table S2**) to sequence the whole exon region of the *DUOX2* gene for patients with *APC* negative polyposis.

### Immunohistochemistry (IHC) staining

IHC staining was performed according to standard protocols. After deparaffinization, antigen retrieval, and blocking, the paraffin-embedded slides were incubated with anti- DUOX2 (1-100 amino acids) antibody (1:500 dilution, ab97266; Abcam, Cambridge, UK), anti-non-phospho (active) β-catenin antibody (1:1,000 dilution; Cell Signaling Technology, Danvers, MA, USA) or anti-GRP78 BiP antibody (1:100 dilution, ab108615; Abcam,). The immunoreactivities were visualized using 3,3’-diaminobenzidine (Dako, Carpenteria, CA, USA).

### Immunofluorescence (IF)

For IF, pretreatment of paraffin-embedded slides was similar to IHC analysis. The slides were incubated simultaneously with rabbit anti-DUOX2 (400–500 amino acids; 1:500 dilution; NB110-61576; Novus Biological, Centennial, CO, USA) and mouse anti-DUOX2 (628–680 amino acids; 1:500 dilution, sc-398681; Santa Cruz Biotechnology, Santa Cruz, CA, USA) antibodies. Cy3-conjugated goat anti-rabbit IgG and 488-conjugated goat anti-mouse IgG (GB21303, and GB25301; Servicebio, Wuhan, China) were used as secondary antibodies. Nuclear counterstaining was performed using 4’,6-diamidino-2-phenylindole. The slide was scanned using the Pannoramic 250/MIDI (3DHISTECH, Budapest, Hungary).

### Cell culture and establishment of cell lines

RKO cells, provided by Stem Cell Bank, Chinese Academy of Sciences (Beijing, China) were grown in Minimum Essential Medium (Gibco, Gaithersburg, MD, USA) supplemented with 10% fetal bovine serum (Gibco), NaHCO_3_ (1.5 g/L), and 1% sodium pyruvate (Invitrogen, Carlsbad, CA, USA).

A 1,588 bp fragment coding hDuox2 K530 with a 6 × His tag was amplified from human *DUOX2* cDNA and was connected to the lentiviral expression vector pLVX-IRES-puro. The plasmid pLVX-IRES-puro-hDuox2-K530 with pMD2.G and pSPAX2 were packaged into viral particles in HEK293T cells. Lentiviral infection was performed according to the manufacturer’s protocols. To obtain a RKO cell line with stable expression of hDuox2 K530, puromycin was used to select the cells. Overexpression was verified by Western blot. Empty vector was transfected in the same way as the negative controls. Plasmids used in the above experiments were all purchased from Youbio (Hunan, China).

### Cell proliferation

Cell Counting Kit-8 (CCK-8; Dojindo, Tokyo, Japan) was used to evaluate cell proliferation. Experiments were performed according to the manufacturer’s instructions. An optical density (OD) value of 450 nm was used to measure cell proliferation. The ratio of the OD value at each time point to the baseline OD value gave the relative proliferation activity. Five replicate wells were set for each group, and 3 independent experiments were performed to calculate the mean and 95% confidence interval (CI).

### Immunocytochemistry

Cells were fixed with 4% paraformaldehyde, permeabilized with 0.1% Triton-X 100 (Sigma-Aldrich, St. Louis, MO, USA), and incubated with anti-6 × His tag (1:500 dilution, MA1-21315; Invitrogen) and rabbit anti-calreticulin (1:400 dilution, 12238; Cell Signaling Technology) antibodies. The cells were then incubated with Alexa Fluor Plus 555-labeled goat anti-mouse IgG (1:200 dilution, A32727; Invitrogen) and Alexa Fluor^®^ 488-labeled anti-rabbit IgG (1:200 dilution, A32731; Invitrogen). Nuclear counterstaining was performed using DAPI.

### *In silico* analysis of missense variants

The functional effects of two missense mutations were predicted using SIFT (version 1.03)^7^, Mutation Taster2^8^, and Mutation assessor^9^ algorithms. The stability change of 2 missense variants were predicted by using the following online available *in silico* algorithms: I-Mutant 2.0^10^, MUpro^11^, and iStable^12^. Three-dimensional (3D) models of the ferric oxidoreductase domain and flavin adenine dinucleotide (FAD)-binding ferredoxin reductase (FR)-type domain of wild-type and mutated hDuox2 were generated using the homology modeling program, SWISS-MODEL^13^. The quality of the 3D models obtained by SWISS-MODEL was verified using the following online available *in silico* algorithms: Verify3.0^14^, PROCHECK^15^, and ProQ^16^. Analysis and visualization of the wild-type and mutated models were performed with the PyMOL visualizer. Default parameters were adopted for all of the above programs. Detailed information about the *in silico* software is summarized in **Supplementary Table S3**.

### Statistical analysis

All statistical analyses were performed using R version 3.6.2 (https://www.r-project.org/). The difference was considered statistically significant when the two-sided *P* value was less than 0.05. Illustrator of biological sequences (IBS) was used to create a schematic of the protein domain^17^. A schematic diagram of the pathogenic mechanism was created using BioRender.com.

## Results

### Identification of *APC* negative polyposis patients

A total of 88 patients suspected of having adenomatous polyposis were enrolled in this study. Using the 139 gene NGS panel and MLPA, we identified 25 unrelated individuals without any specific *APC* pathogenic variants, named *APC* negative polyposis. The clinical characteristics, including the age at first visit, sex, FAP classification, extra-intestinal manifestation, and the family history of patients with *APC* negative and *APC* mutant polyposis are summarized in **Supplementary** **Table S4**. Consistent with our expectations, patients with *APC* negative polyposis were diagnosed at a later age, and had a lighter polyp load, with fewer extra-intestinal manifestations and a family history related to polyposis.

### Identification of 3 unrelated individuals with *DUOX2* variants

**Figure 2:**
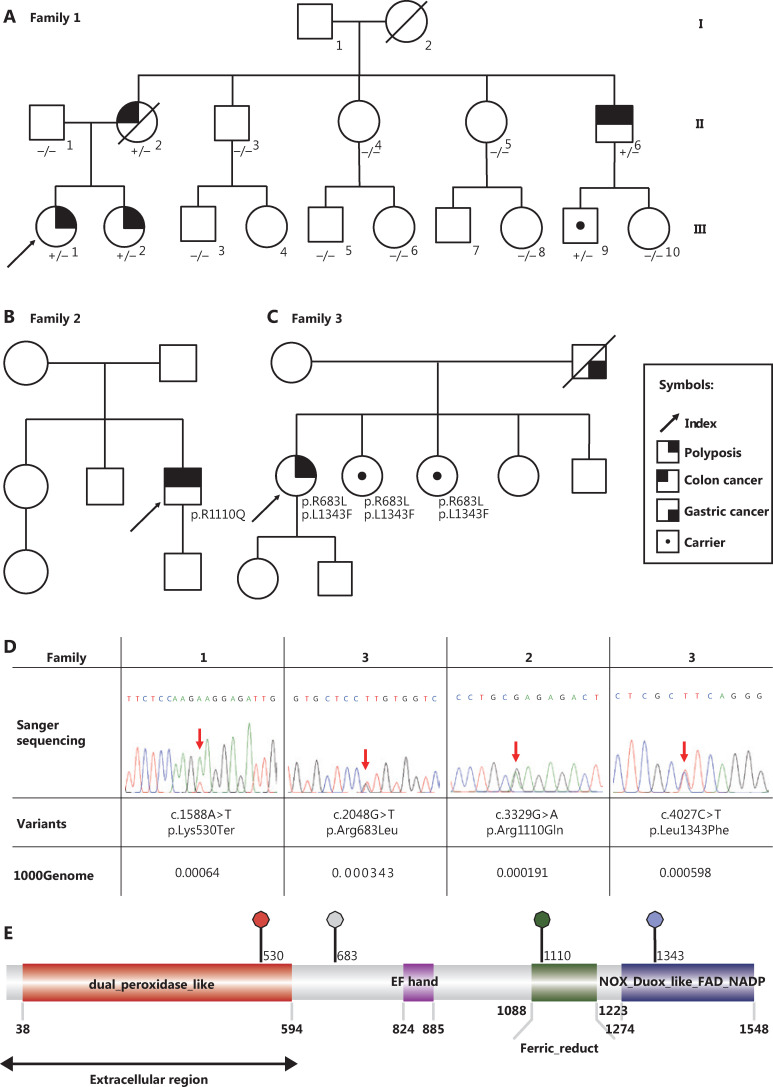
Pedigrees and variants of 3 unrelated families carrying *DUOX2* variants. Pedigrees of family 1(A), 2(B), and 3(C). The genotype and phenotype information are displayed, +/− represents a heterozygous nonsense (c.1588A>T; p.K530X) variant in the *DUOX2* gene, and −/− represents the wild type. (D) The four *DUOX2* variants in these 3 families were validated by Sanger sequencing. Their locations are depicted in a corresponding lollipop plot of *DUOX2* (created with IBS) and the conserved domains of the hDuox2 protein are also shown (E).

We performed WES on one of the families with *APC* negative polyposis (the pedigree is shown in **Figure 2A**). The proband (III-1 in **Figure 2A**) was a female with 20–30 tubulovillous adenomas in the proximal colon at the age of 26 years. Meanwhile, her younger sister (III-2) also underwent colonoscopy, which revealed many polyps at the age of 24 years. She subsequently underwent total colectomy with ileorectal anastomosis. Pathology confirmed hundreds of adenomas scattered over the colon, mainly in the right half. Their mother (II-2) died from colon cancer with distant metastases at the age of 47 years, but the history of polyps in the intestine was uncertain. During our follow-up, one of their mother’s brothers (II-6) underwent an endoscopy revealing an ascending colon adenocarcinoma and dozens of adenomas in the sigmoid colon at the age of 51 years.

The reasons for choosing this family were as follows: 1. four patients (II-2, II-6, III-1, and III-2) with colorectal adenomatous polyposis or colorectal cancer diagnosed in the family; 2. III-2 had a classic polyposis phenotype (> 100 polyps); and 3. we were able to obtain a complete genetic sample to help analyze the co-segregation of the family. WES showed that a total of 17,654 variants were shared by 3 patients (II-2, III-1, and III-2), and 2 unaffected individuals (II-1 and II-4) did not carry them. Synonymous or intronic variants other than those affecting consensus splice sites were excluded from further analysis. According to the American College of Medical Genetics and Genomics standards and guidelines, we selected the pathogenic or the likely pathogenic variants, and excluded the high occurrence variants (> 0.1% in the 1000 Genomes database). We identified a heterozygous nonsense variant c.1588A>T; p.K530X in the *DUOX2* gene, which might have been critical to pathogenesis in this family with *APC* negative polyposis.

By sequencing the whole exon region of the *DUOX2* gene from other patients with *APC* negative polyposis using Sanger sequencing, we additionally found 2 unrelated patients (pedigrees are shown in **Figure 2B and 2C**) carrying missense variants in the *DUOX2* gene. The Sanger validation is shown in **Figure 1D**, and the 2 missense variants (p.R1110Q and p.L1343F) were both located in highly conserved protein domains (**Figure 2E**).

### The effect of truncated proteins from the *DUOX2* nonsense variant on polyposis

#### The hDuox2 is overexpressed in adenomas independent of the Wnt pathway

**Figure 3:**
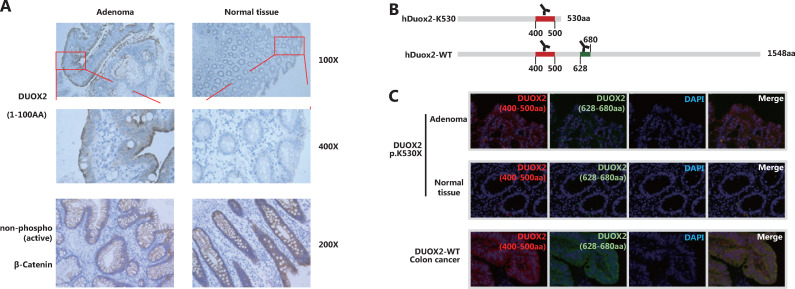
The truncated protein, hDuox2 K530, is overexpressed in adenomas. (A) Immunohistochemistry (IHC) staining of hDuox2 with an antibody targeting the N-terminal 1–100 amino acids of human Duox2 protein in the adenoma and normal tissues from a *DUOX2* p.K530X carrier. The expression of non-phospho (active) β-Catenin was also detected in these 2 samples by IHC analysis. (B) The positions of the amino acid sequence recognized by 2 different anti-Duox2 antibodies are marked. (C) Immunofluorescence staining of wild-type and truncated proteins of hDuox2 in adenomas and normal tissues from a *DUOX2* p.K530X carrier as well as the tumor from a *DUOX2* wild-type patient. AA, amino acid.

We obtained paraffin-embedded adenoma and adjacent normal tissues from the affected case (III-2 in Family 1). IHC staining with an antibody targeting 1–100 amino acids of Duox2 revealed that its expression level in adenoma tissue was significantly higher than that in normal colon mucosa (**Figure 3A**). In addition, we also found that there was no obvious difference in the expression of non-phospho (active) β-catenin between the 2 samples, which indicated that the Wnt pathway was not activated abnormally in this adenoma tissue.

#### Truncated protein hDuox2 K530 was overexpressed in the adenoma, instead of the wild-type protein

In the patient that was a heterozygous carrier, the adenoma was able to express both wild-type (hDuox2-WT) and truncated proteins (hDuox2 K530) of *DUOX2*. However, the antibodies used in IHC analysis could bind to both these proteins, making it impossible to determine which protein was overexpressed in the adenoma. To overcome this issue, IF analysis was conducted using two antibodies with different immunogenicities (**Figure 3B**) to determine whether the wild-type protein or the truncated protein was overexpressed. The antibody targeting 400–500 amino acids of hDuox2 enabled the detection of both wild-type and truncated proteins, and another antibody targeting 628–680 amino acids could only bind to the wild-type hDuox2. In the FFPE samples from the *DUOX2* p.K530X carrier (III-2 in Family 1), normal colon mucosa did not react with both antibodies, which was consistent with the results observed by IHC analysis. Moreover, we found that adenoma tissue only showed positive results with the antibody targeting 400–500 amino acids, but not with another antibody, indicating that the truncated protein, hDuox2 K530, was overexpressed in the adenoma. In addition, the tumors from patients with *DUOX2* wild-type colon cancer served as a positive control (**Figure 3C**).

#### Overexpression of the truncated protein hDuox2 K530 activates cell proliferation through the unfolded protein response

**Figure 4:**
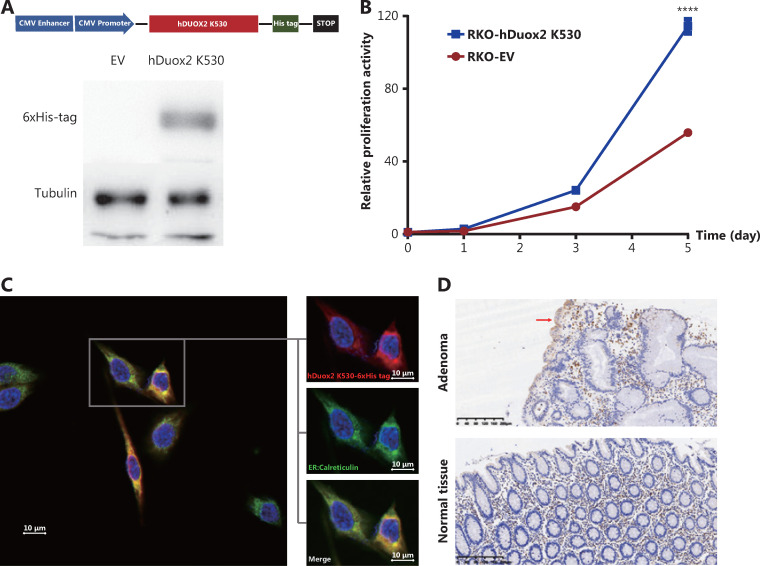
Overexpression of truncated protein, hDuox2 K530, promotes cell proliferation through the unfolded protein response. (A) Construction of the RKO cell line with stable expression of hDuox2 K530. (B) Growth curve of RKO-hDuox2 K530 and the control group. ^****^P < 0.0001 (C) Confocal microscopy with anti-6 × His-tag (red) and anti-calreticulin (green) in the RKO-hDuox2 K530 cell line. (D) Immunohistochemistry staining (100×) of GRP78 in the adenoma and normal tissues from a DUOX2 p.K530X carrier. EV, empty vector.

To determine the effect of the truncated protein on polyposis, we generated a human colorectal cancer cell line, RKO, stably overexpressing hDuox2 K530 with a 6 × His tag (**Figure 4A**). CCK-8 analysis showed that from day 3 to day 5, the relative absorbance of RKO-hDuox2 K530 was significantly higher than that of the control group (*P* < 0.0001; **Figure 4B**). This indicated that overexpression of truncated protein could cause abnormal cell proliferation, which explained the occurrence of adenomas. Regarding the underlying mechanism, we hypothesized that the truncated protein could not be folded into a higher level protein structure due to the incomplete amino acid sequence, and was therefore retained in the endoplasmic reticulum (ER)^18^. The continuous expression of truncated proteins activated the unfolded protein response (UPR) of the ER, resulting in abnormal cell proliferation^19^. Through IHC, we observed that the truncated protein was co-localized with the ER marker, calreticulin (**Figure 4C**). Moreover, the expression level of GRP78 (BiP, one of the UPR markers^20^) increased in adenomas from the *DUOX2* p.K530X carrier, which preliminarily confirmed our hypothesis (**Figure 4D**).

### *In silico* analysis of two *DUOX2* missense variants

**Figure 5:**
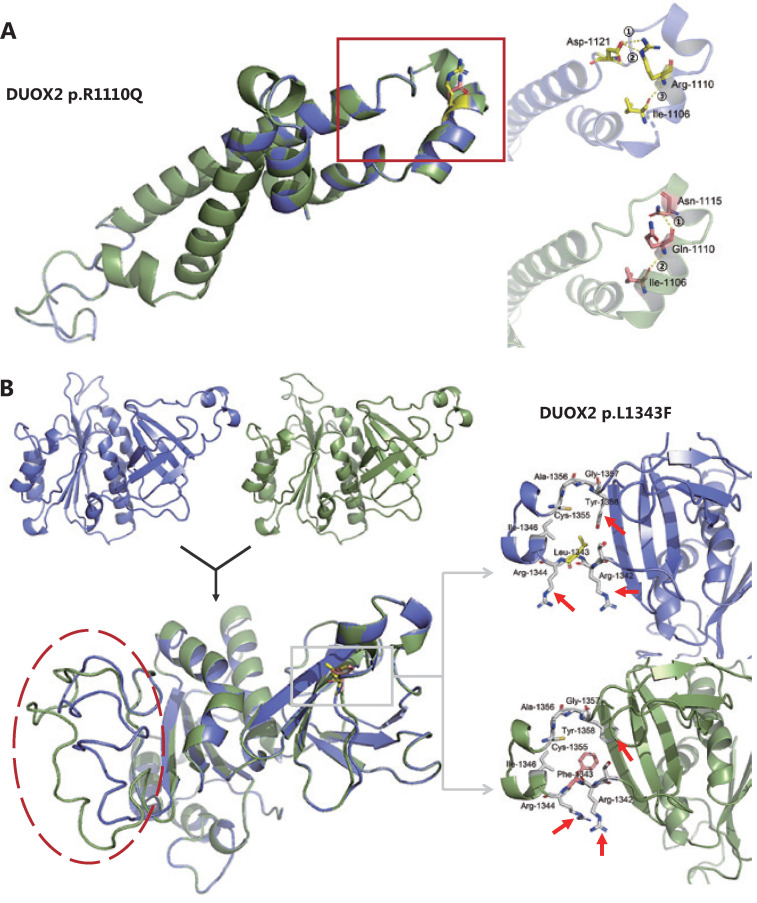
Three-dimensional (3D) models of the wild-type (blue) and mutant (green) domains. (A) Superimposition of the 3D model between wild-type (blue) and mutant (green) Ferric oxidoreductase domains are displayed. A magnified view of the region containing the wild-type Arg1110 residue (yellow) and its hydrogen bonds (yellow) formed with other residues is shown in the right upper panel. The magnified view of the mutant Gln1110 residue (pink) is shown in the right lower panel. (B) The 3D models and the superimposition between wild-type (blue) and mutant (green) FAD-binding FR-type domains are displayed. Magnified views of the region containing the wild-type Leu1343 residue (yellow) and the mutant Phe1343 residue (pink) are shown in the right upper and lower panels, respectively. Note that 3 residues around the mutation site have changed directions (red arrow).

We identified 3 missense variants in the *DUOX2* gene (p.R683L, p.R1110Q, and p.L1343F) from 2 unrelated families with unexplained polyposis. Because the variant p.R683L did not occur in the conserved domain, we conducted an *in silico* analysis on the other 2 variants. First, 3 different online servers (SIFT, Mutation Taster2, and Mutation assessor) based on multiple algorithms, were used for structural and functional annotation of these 2 missense variants (results are shown in **Table 1**). Next, stability analysis was conducted using I-Mutant2.0, MUpro, and iStable. Both variants were predicted to decrease protein stability. Finally, we performed 3D modeling analysis of the wild-type and mutant domains (**Figure 5**). As for the variant p.R1110Q, we found that in the wild-type hDuox2 protein, a network of 3 intrahelical hydrogen bonds existed between Arg1110 and the other 2 residues, Ile1106 and Asp1121. When the Arg1110 was mutated to Gln due to a mutation, the 2 hydrogen bonds between Arg1110 and Asp1121 were lost and replaced by a novel hydrogen bond with Asn1115, which could influence the stability of the α-helix (**Figure 5A**). Additionally, comparing tertiary structures of the wild-type and mutant caused by p.L1343F, we observed that the directions of the amino acid molecules around the mutation site changed slightly (arrows in **Figure 5B**), and distant random coils showed a significant difference between the 2 domains (red circle in **Figure 5B**).

**Table 1:** Prediction of effects of missense variants on protein function and stability

Variant	Function prediction			Stability prediction					
	SIFT^†^	Mutation Taster2^‡^	Mutation assessor^§^	i-Mutant2.0^¶^		MUpro^††^		iStable^‡‡^	
				Prediction	DDG value	Prediction	Conf. score	Prediction	Conf. score
c.3329G > A (p.Arg1110Gln)	Deleterious	Disease causing		Decrease	−1.02	Decrease	−0.68	Decrease	0.72
c.4027C > T (p.Leu1343Phe)	Deleterious	Disease causing	Medium	Decrease	−1.23	Decrease	−1	Decrease	0.87

^†^SIFT classifies an amino acid substitution as “deleterious” or “tolerated” according to the normalized probability. A substitution is predicted as deleterious if the score is < 0.05 and tolerated if the score is ≥ 0.05.

^‡^Mutation Taster2 predicts an alteration as one of 4 possible types: “disease causing,” “disease causing automatic”, “polymorphism,” and “polymorphism automatic.”

^§^Mutation assessor outputs the annotations of functional impact of a variant as functional (high, medium), predicted non-functional (low, neutral).

^¶^I-Mutant2.0 outputs free energy change value (DDG) between mutant and wild-type protein. Stability decreases if DDG < 0 and increases if DDG > 0.

^††^MUpro uses a confidence score between -1 and 1 to predict the effects of mutation on protein stability and measure the confidence of the prediction. A score < 0 means the mutation decreases the protein stability, and the smaller the score, the more confident the prediction. Conversely, a score > 0 means the mutation increases the protein stability, and the bigger the score, the more confident the prediction.

^‡‡^iStable outputs the prediction as “stability decrease” or “stability increase.”

## Discussion

*APC* negative polyposis refers to patients with clinical features of FAP, but conventional genetic testing, such as NGS and MLPA, failed to define their pathogenic variants. The causes may include the following 3 aspects. 1. Some variants in the non-coding region of the* APC* gene, such as the promoter region^21^, could be excluded by NGS, which only covers the exon regions as well as the first and last 20 bps of the intron regions. 2. No genetic factors, but some environmental factors or personal habits cause polyps in the intestine^22^. 3. There are other genes with pathogenic variants, even those that have never been reported to be associated with polyposis. Pathogenic genes related to adenomatous polyposis are mainly divided into these 2 fields. First, the loss-of-function of key components in the Wnt pathway causes abnormal activation, such as *APC*^3^ and *AXIN2*^23^. Second, several repair-related genes during DNA replication are also thought to be associated with polyposis, such as *MUTYH*^4^, *NTHL1*^24^, *MSH3*^25^, *POLE*, and *POLD1*^26–28^.

This is the first study to report that germline variants of the *DUOX2* gene could also be implicated in a fraction of adenomatous polyposis cases. Dual oxidase 2 (Duox2) is one of the important members of the nicotinamide adenine dinucleotide phosphate (NADPH) oxidase family. It forms a complex with its maturation factor, Duoxa2, to catalyze the synthesis of hydrogen peroxide (H_2_O_2_). At first, Duox2 was thought to be active only in the thyroid, being involved in thyroid hormone synthesis^29^. Later, Duox2 was found to be expressed in various tissues, including the entire intestinal mucosa^30^. Most importantly, Duox2 acts as the first barrier of the intestinal epithelium and participates in the innate immune response of intestinal mucosa to maintain immune homeostasis by producing H_2_O_2_^31, 32^. Previous studies have reported that several missense variants (p.R1211C, p.R1492C. and p.P303R) of *DUOX2* are thought to be crucial in some patients with inflammatory bowel disease (IBD), especially those with very early onset IBD^33–35^. In recent years, there have been several studies on the role of *DUOX2* in cancer, especially colon cancer, suggesting that *DUOX2* is an oncogene that facilitates metastatic ability^36^ and chemoresistance^37^ of CRC cells. However, there is no published report indicating the association of *DUOX2* germline variants with adenomatous polyposis. After an extensive literature review, we found that in 2006, a Japanese research team had already observed that the expression of Duox2 in flat polyps was more than 10 times higher than in the adjacent normal epithelial tissues^38^. This suggested that overexpression of Duox2 might be an important factor in the occurrence of polyps.

Duox2 protein is a 7-transmembrane protein composed of an N-terminal peroxidase-like domain (outside the cell), 2 EF-hand domains, and a C-terminal region binding to NADPH and FAD (inside the cell)^39^. Under physiological conditions, immature and partially glycosylated Duox2 is in the ER and is transported from the ER to the Golgi apparatus during certain types of stimulations^40^. After processing and glycosylation, it is finally localized on the cell membrane, acting as a transmembrane protein. Due to the nonsense variant, p.K530X, the translation of the Duox2 protein is terminated at the Tyr530. IHC and IF assays also confirmed that the truncated protein, hDuox2 K530, was overexpressed in an adenoma from a heterozygous variant carrier. As it contains only the N-terminal peroxidase-like domain, the truncated protein cannot be processed and modified as expected; thus, it is retained in the ER. We found that the truncated protein was located in the ER of RKO-hDuox2 K530 cells. The high expression level of hDuox2 K530 caused ER stress and adversely affected cells, leading to the activation of the UPR to remove these adverse effects, maintaining cell stability by stopping protein translation, improving folding ability, degrading abnormally folded protein, or eventually inducing an apoptosis state^41^. During this time, the expression of GRP78, a UPR marker, would be significantly upregulated^20^, and we were able to detect this change by IHC staining of the adenoma from the patient.

However, some studies have suggested that continued excessive activation of the UPR is closely related to the occurrence and development of tumors, including promoting cell proliferation and migration, inducing DNA damage and mediating immune escape^19^. Activating transcription factor 6 (ATF6) and its downstream pathways play a key role after UPR activation^42^. Coleman et al.^43^ observed that 12-week-old transgenic nATF6^IEC^ mice spontaneously developed adenomatous polyps in the intestine without being stimulated. We also found that the RKO-hDuox2 K530 cell line had significantly faster cell proliferation than the control group. Whether this was due to the abnormal activation of the UPR and its downstream pathways remains to be clarified.

There were 6 affected individuals in the 3 pedigrees with *DUOX2*-associated polyposis. Considering the clinical features, we summarized the following points. First, if it is not diagnosed early, the number of polyps can exceed 100 and may develop into malignant tumors. However, if diagnosed and treated in the early stage of the disease, the prognosis of the patient is significantly better than that of the FAP. For example, the first index (III-1 in Family 1) has been followed-up since the initial diagnosis in December 2017. After the 20–30 polyps were removed under the colonoscope, only a new 0.2 cm inflammatory polyp was noted during regular review. Second, none of these cases had extracolonic manifestations associated with FAP, such as congenital hypertrophy of the retinal pigment epithelium and desmoid tumor. Finally, through genetic testing of the members in families 1 and 3, we found that the penetrances of the *DUOX2* were 80% and 33.3%, respectively. We believe that the penetrance may depend on the type and location of the variants.

This study had several limitations. Due to the small population size, we found only 3 *DUOX2*-related polyposis families. Although bioinformatics tools were used to analyze the potential pathogenicity of the missense variants, we failed to explain the pathogenic mechanisms through functional experiments, and we could not adequately summarize the clinical characteristics of those patients. Thus, it is necessary to include the *DUOX2* gene in the genetic screening for adenomatous polyposis and to collect more patients with *DUOX2*-related polyposis in a larger population.

## Conclusions

**Figure 6:**
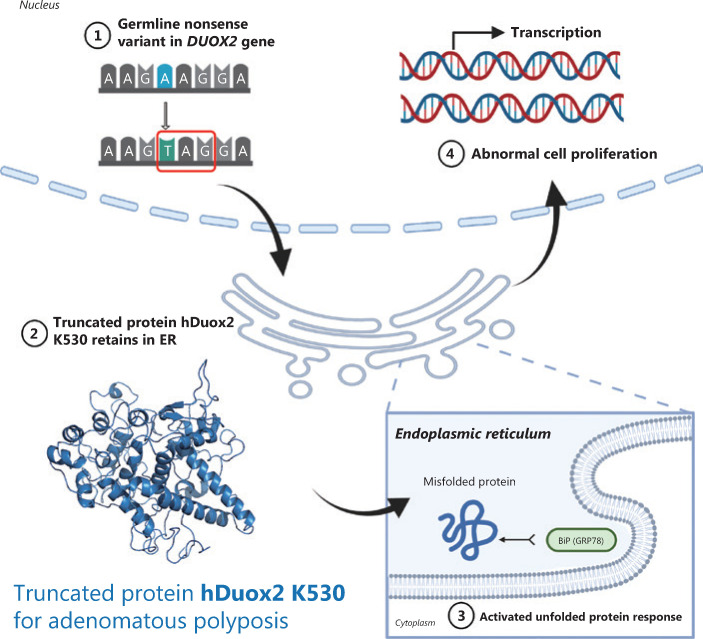
Schematic diagram of truncated protein, hDuox2 K530, causing adenomatous polyposis. The diagram shows that the truncated protein, hDuox2 K530, is translated due to a nonsense variant (c.1588A>T) occurring in the *DUOX2* gene. Therefore, this misfolded protein is retained in the endoplasmic reticulum and activates an unfolded protein response, which could induce abnormal cell proliferation.

In conclusion, by evaluating patients with *APC*-negative polyposis, we conducted WES and Sanger sequencing to identify three unrelated patients carrying *DUOX2* germline variants, suggesting that the *DUOX2* gene might be a novel disease-associated gene for adenomatous polyposis with an autosomal dominant inheritance. Based on a literature search and functional experiments, we speculate that the mutant protein causes adenoma by activating the UPR in the ER (**Figure 6**). Further studies are required to validate this possibility.
