# Erratum to A truncated protein product of the germline variant of the *DUOX2* gene leads to adenomatous polyposis

**DOI:** 10.20892/j.issn.2095-3941.2021.1213

**Published:** 2022-01-26

**Authors:** Mengyuan Yang, Yingxin Zhao, Yuwei Ding, Juan Wang, Yinuo Tan, Dong Xu, Ying Yuan

**Affiliations:** 1 Department of Medical Oncology, Key Laboratory of Cancer Prevention and Intervention, Ministry of Education, The Second Affiliated Hospital, Zhejiang University School of Medicine, Hangzhou 310009, China; 2 Cancer Institute, Key Laboratory of Cancer Prevention and Intervention, Ministry of Education, The Second Affiliated Hospital, Zhejiang University School of Medicine, Hangzhou 310009, China; 3 Department of Colorectal Surgery and Oncology, Key Laboratory of Cancer Prevention and Intervention, Ministry of Education, The Second Affiliated Hospital, Zhejiang University School of Medicine, Hangzhou 310009, China

In the published paper^1^, the author’s organization information on page 215 contained errors. The author information has been updated to correct the errors. The errors do not affect the conclusions of this article. We apologize for the errors and for any confusion they may have caused.
