# Supplementary material for: A truncated protein product of the germline variant of the DUOX2 gene leads to adenomatous polyposis
Source: Cancer Biol Med. 2021 Feb 15;18(1):215–26. doi: 10.20892/j.issn.2095-3941.2020.0305 (PMC7877186; doi:10.20892/j.issn.2095-3941.2020.0305)
Supplement: Supplementary file 1 [file cbm-18-215-s001.pdf]

Supplementary materials

Table S1 139 genes detected *via* next-generation sequencing (NGS)

AIP	***ALK***	***APC***	***ATM***	***ATR***	***AXIN2***	***BAP1***	***BARD1***
BLM	***BMPR1A***	***BRCA1***	***BRCA2***	*BRIP1*	*BUB1B*	*CBL*	*CDC73*
CDH1	*CDK4*	*CDKN1B*	*CDKN1C*	*CDKN2A*	*CEBPA*	*CHEK1*	***CHEK2***
CYLD	*DDB2*	*DICER1*	*DIS3L2*	*EGFR*	*ELANE*	***EPCAM***	*ERCC1*
ERCC2	***ERCC3***	***ERCC4***	***ERCC5***	*EXT1*	*EXT2*	*EZH2*	*FANCA*
FANCB	*FANCC*	*FANCD2*	*FANCE*	*FANCF*	*FANCG*	*FANCI*	*FANCL*
FANCM	*FAS*	*FH*	*FLCN*	GALNT12	*GATA2*	*GEN1*	*GJB2*
GPC3	***GREM1***	*HMBS*	*HNF1A*	*HOXB13*	*HRAS*	***KIT***	*LASP1*
MAX	*MC1R*	*MEN1*	*MET*	*MITF*	***MLH1***	***MLH3***	*MRE11A*
MSH2	***MSH6***	*MTAP*	*MTUS1*	***MUTYH***	*NBN*	*NF1*	*NF2*
NSD1	*NTRK1*	*PALB2*	*PALLD*	***PDGFRA***	*PHOX2B*	***PMS1***	***PMS2***
POLD1	***POLE***	***POLH***	*PPM1D*	*PRKAR1A*	*PRSS1*	*PTCH1*	*PTCH2*
PTEN	*PTPN11*	*RAD50*	*RAD51B*	*RAD51C*	*RAD51D*	*RB1*	*RECQL*
RECQL4	*RET*	*RHBDF2*	*RUNX1*	*SBDS*	*SDHA*	*SDHAF2*	***SDHB***
SDHC	***SDHD***	*SLX4*	***SMAD4***	*SMARCA4*	*SMARCB1*	*SMARCE1*	*SOSO1*
STAT3	***STK11***	*SUFU*	*TERT*	*TGFBR1*	*TMEM127*	***TP53***	*TSC1*
TSC2	*UROD*	*USHBP1*	*VEGFA*	*VHL*	***WRN***	*WT1*	***XPA***
XPC	*XRCC2*	*ZMAT3*					

Genes with bold font are currently reported to be related to tumors in the digestive tract.

Table S2 Primers used to sequence the whole exon region of the *DUOX2* gene

Exons	Primer-F	Primer-R
**1**	5′-TTGGCGTCTCTTTGCGTACA-3′	5′-CTTCTGGGGCATGTCAGTCC-3′
**2-3**	5′-CAGTCAGGACGCACTCTCAC-3′	5′-TCCAGTGACACCCCTAGGTT-3′
**4**	5′-TGCCTACACAGCGCAATCTT-3′	5′-CAGTCTCGAAGTGCTGCGTA-3′
**5-6**	5′-ATTGAGGCTACCTGGGGCTAAC-3′	5′-GCGATGACCCTCTTGCGT-3′
**7-9**	5′-GCCATCTATGGCTCCTCGCA-3′	5′-AGAGTGCGCTCCTAGTCCAG-3′
**10-11**	5′-GGCTGAAATTTGGTGGCTGG-3′	5′-GGTACCACCCCTCCCCAATA-3′
**12**	5′-CAGCATGGGCACAGATCTCA-3′	5′-CCTACCCACGGTAACACCAC-3′
**13**	5′-GGGGGCTTGCCTAAGAAGAG-3′	5′-GGTTGGGAAGGGTGTGGTG-3′
**14**	5′-GGTTTCACTATGCTGGTCAGGC-3′	5′-AGGCCCACCTCCCTGAAACTG-3′
**15**	5′-ATCAAGATTGAGGGTGCGGG-3′	5′-CTGGCACTCCATCTTTGGCT-3′
**16**	5′-CACCCTGCCCTCAACCTAAG-3′	5′-GGGATAATTGGGCCGGGTAG-3′
**17**	5′-CCAAAGATGGAGTGCCAGGT-3′	5′-AGCCTCCCCTCAATGGATCT-3′
**18**	5′-ATCACAAGCGAGGCCACCC-3′	5′-CATAGAGCGGAAGCTTAGTTCAC-3′
**19-20**	5′-AGAGATTTGATTCCCCCGTCT-3′	5′-TTGTCGTTGGCCATATTGGTG-3′
**21-22**	5′-TTCTCTGATTGGTCAAGGTCACTT-3′	5′-CCAAGCATCCGAGCAGCATAG-3′
**23-24**	5′-CCTATGCTGCTCGGATGCTT-3′	5′-AGGCAAAGCCATAGTCTGGG-3′
**25**	5′-TGGCAATCTTCTCGGCCATC-3′	5′-AAACCATCCCCAGAACTGACC-3′
**26**	5′-CTTGGGTTCATGCCATTCTCC-3′	5′-CTCCTCCCTATGCCTCCTCTC-3′
**27-29**	5′-GAAAAATTGCTGAGTACAGAGGGTG-3′	5′-TGCAAGTCTCCTGGGAGCTAA-3′
**30-31**	5′-AGCTCTTGCATTACCTGGGAAAC-3′	5′-CCACTGGGTAAGAATGACCCCT-3′
**32-34**	5′-GGTACCACCCCTCCCCAATA-3′	5′-CTTCTTTGGGAGATCCTGACTGG-3′

Table S3 Information of *in silico* algorithms

Type	*In-silico* algorithm	Website	Introduction
Prediction of function effect	SIFT	http://sift.jcvi.org/	SiFT is a sequence homology-based tool that predict tolerated and deleterious substitutions for every position of the query sequence by using multiple alignment information
	MutationTaster2	http://www.mutationtaster.org/	MutationTaster2 is a web-based software to predict the functional consequences of not only amino acid substitutions but also intronic and synonymous alterations, short insertion and/or deletion (indel) mutations and variants spanning intron-exon borders
	Mutation assessor	http://mutationassessor.org/r3/	Mutation assessor predicts the functional impact of amino-acid substitutions in proteins, based on evolutionary conservation of the affected amino acid in protein homologs
Prediction of stability effect	i-Mutant2.0	http://folding.biofold.org/i-mutant/i-mutant2.0.html	I-Mutant 2.0 is a Support Vector Machine-based tool that predicts the protein stability change upon single point single-site mutations, and was trained on a data set derived from ProTherm
	Mupro	http://mupro.proteomics.ics.uci.edu/	MUpro is a set of machine learning programs using Support Vector Machines and Neural Networks to predict how single-site amino acid mutation affects protein stability
	iStable	http://predictor.nchu.edu.tw/iStable/	iStable is an integrated predictor constructed by using sequence information and prediction results from different element predictors
Modeling	SWISS-MODEL	https://swissmodel.expasy.org/	SWISS-MODEL is a fully automated protein structure homology-modelling server
Verification of model's quality	Verify3.0	https://servicesn.mbi.ucla.edu/Verify3D/	Verify3D works by assigning a structural class based on its location and environment to determine the compatibility of an atomic model (3D) with its own amino acid sequence (1D) and comparing the results to good structures
	PROCHECK	http://www.csb.yale.edu/userguides/datamanip/procheck/manual/index.html	PROCHECK assess how normal or unusual the geometry of the residues in a given protein structure is, as compared with stereochemical parameters derived from well-refined, high-resolution structures
	ProQ	https://proq.bioinfo.se/cgi-bin/ProQ/ProQ.cgi	ProQ is a neural network based predictor that based on a number of structural features predicts the quality of a protein model

Table S4 Clinical characteristics of *APC* negative FAP patients

Characteristics	Overall (*n* = 89)	*APC*-negative FAP (*n* = 25)	*APC*-mutant FAP (*n* = 64)	P^a
Age of first visit		45.9 ± 14.6	31.7 ± 9.41	<0.001
Gender				ns
Male	43	12 (46.2%)	31 (49.2%)	
Female	46	14 (53.8%)	32 (50.8%)	
FAP classification[b]				<0.001
Classic	50	7 (26.9%)	43 (68.3%)	
Attenuated	39	19 (73.1%)	20 (31.7%)	
Extraintestinal manifestations[c]				0.009
Without	55	22 (84.6%)	33 (52.4%)	
With	22	4 (15.4%)	18 (28.6%)	
Not sure	12	0 (0%)	12 (19.0%)	
Family history[d]				<0.001
No family history	37	13 (50.0%)	24 (38.1%)	
Polyposis	38	4 (15.4%)	34 (54.0%)	
Other cancers	14	9 (34.6%)	5 (7.9%)	

[a]*P* values obtained from the *t*-tests (continuous variables) and χ^2 tests (categorical variables).
[b]The patients with more than 100 polyps in the intestinal tract are classified as classic familial adenomatous polyposis (FAP) while 10–99 are attenuated FAP.
[c]Extraintestinal manifestations include gastric polyps, congenital hypertrophy of the retinal pigment epithelium (CHRPE) and desmoid tumor.
[d]Family history was coded as no family history [no affected first-degree relatives (FDRs)], polyposis (for polyposis, at least one affected FDR), other cancers (for other cancers, like colorectal cancer, at least one affected FDR).
